# Predictive Factors for Anastomotic Leakage After Colorectal Surgery: Study Protocol for a Prospective Observational Study (REVEAL Study)

**DOI:** 10.2196/resprot.5477

**Published:** 2016-06-09

**Authors:** Audrey CHM Jongen, Joanna WAM Bosmans, Serdar Kartal, Tim Lubbers, Meindert Sosef, Gerrit D Slooter, Jan H Stoot, Frederik-Jan van Schooten, Nicole D Bouvy, Joep PM Derikx

**Affiliations:** ^1^Department of General SurgeryMaastricht University Medical CentreMaastrichtNetherlands; ^2^NUTRIM School for Nutrition & Translational Research in MetabolismMaastricht UniversityMaastrichtNetherlands; ^3^Department of SurgeryMaxima Medical CentreVeldhovenNetherlands; ^4^Department of SurgeryZuyderland Medical CentreHeerlenNetherlands; ^5^Department of SurgeryZuyderland Medical CentreSittardNetherlands; ^6^Pediatric Surgical Centre AmsterdamEmma Children's hospitalAmsterdam Medical Centre/Vrije Universiteit Medisch CentrumAmsterdamNetherlands

**Keywords:** Anastomotic leakage, Colorectal surgery, Postoperative complications, Biomarkers, Personalized medicine

## Abstract

**Background:**

Anastomotic leakage (AL) remains the most important complication following colorectal surgery, and is associated with high morbidity and mortality rates. Previous research has focused on identifying risk factors and potential biomarkers for AL, but the sensitivity of these tests remains poor.

**Objective:**

This prospective multicenter observational study aims at combining multiple parameters to establish a diagnostic algorithm for colorectal AL.

**Methods:**

This study aims to include 588 patients undergoing surgery for colorectal carcinoma. Patients will be eligible for inclusion when surgery includes the construction of a colorectal anastomosis. Patient characteristics will be collected upon consented inclusion, and buccal swabs, breath, stool, and blood samples will be obtained prior to surgery. These samples will allow for the collection of information regarding patients’ inflammatory status, genetic predisposition, and intestinal microbiota. Additionally, breath and blood samples will be taken postoperatively and patients will be strictly observed during their in-hospital stay, and the period shortly thereafter.

**Results:**

This study has been open for inclusion since August 2015.

**Conclusions:**

An estimated 8-10% of patients will develop AL following surgery, and they will be compared to non-leakage patients. The objectives of this study are twofold. The primary aim is to establish and validate a diagnostic algorithm for the pre-operative prediction of the risk of AL development using a combination of inflammatory, immune-related, and genetic parameters. Previously established risk factors and novel parameters will be incorporated into this algorithm, which will aid in the recognition of patients who are at risk for AL. Based on these results, recommendations can be made regarding the construction of an anastomosis or deviating stoma, and possible preventive strategies. Furthermore, we aim to develop a new algorithm for the post-operative diagnosis of AL at an earlier stage, which will positively reflect on short-term survival rates.

**Trial Registration:**

Clinicaltrials.gov: NCT02347735; https://clinicaltrials.gov/ct2/show/NCT02347735 (archived by WebCite at http://www.webcitation.org/6hm6rxCsA)

## Introduction

Surgery remains the predominant curative treatment for patients with colorectal cancer (CRC), but can lead to severe post-operative complications, of which anastomotic leakage (AL) is the most feared. AL occurs in 8-15% of patients undergoing colorectal surgery and is associated with high morbidity and short-term mortality rates of up to 39% [1-4]. AL often requires one or more re-interventions, leading to a significant increase in length of hospital stay and subsequently to high health care costs [3]. Some authors even suggest that AL is associated with an impaired oncological prognosis [5-8]. Both decreased disease-specific survival (odds ratio [OR] 1.75) [9,10] and an increased local recurrence rate of CRC (OR 2.9) [10] have been reported.

In the past decades, important risk factors for AL such as male gender [4], neo-adjuvant chemotherapy [11], tumor size [12], malnutrition [13], smoking [14], steroid treatment [1,15], and the use of non-steroidal anti-inflammatory drugs (NSAIDs) [16] have been identified. Despite the determination of these risk factors for AL, surgeons’ prediction of the risk to develop AL for the individual patient remains inaccurate [17]. Despite decades of extensive research on preventive methods, the introduction of innovative surgical techniques, and fast-track protocols, incidence rates of AL have remained stable [18].

A recently conducted study in one of the participating centers, that can be considered a pilot study, suggests that pre-operative levels of a certain plasma marker (intestinal fatty acid binding protein, I-FABP) can predict the development of AL in patients undergoing CRC surgery. I-FABP is present in mature enterocytes of the small and large intestine, and is released upon intestinal damage [19]. It is hypothesized that increased plasma levels of I-FABP are the result of an underlying clinical condition of the gastrointestinal tract, predisposing the patient to AL [20].

Cumulative evidence in the literature suggests that perioperative pain treatment with NSAIDs, commonly prescribed analgesics that inhibit cyclo-oxygenase 2 (COX-2) expression, increase the risk of AL [16,21,22]. COX-2 knockout mice have an increased risk of developing AL, and this risk can be reduced by the administration of prostaglandin E2, a product of COX-2 [23]. Another study conducted by our research group has demonstrated that decreased COX-2 expression, due to a polymorphism in the COX-2 gene, leads to an increased risk of the development of AL [23]. Furthermore, it has been shown that patients with different genotypes of mannose-binding lectin (MBL), an important complement factor of the immune system, respond differently to intestinal damage [24].

In the pilot study mentioned previously, it was shown that a combination of C-reactive protein (CRP) and calprotectin levels in plasma provides high diagnostic accuracy for AL during the post-operative period. Finally, we have shown that functional compromise (characterized by malnutrition, frailty, and sarcopenia) is associated with post-operative morbidity and mortality [25]. Malnutrition, as indicated by high Short Nutritional Assessment Questionnaire (SNAQ) scores, and sarcopenia were predictive for, or showed a trend towards, prediction of AL.

Based on previous literature and both our experimental and clinical data, we hypothesize that the occurrence of AL is partly due to patient-derived factors such as a compromised immune response, sarcopenia, genetic predisposition, and an aberrant intestinal microbiome composition, and that the post-operative course can be further influenced by surgical stress, ischemia, and a derailed systemic response. Our hypothesis is that, based on these parameters, individual risks for the development of AL can be assessed pre-operatively.

Besides the obvious improvements that can be made regarding pre-operative risk assessment, the post-operative recognition and management of AL has also proven to be challenging. The timing of AL diagnoses varies greatly, from post-operative day 3 to beyond 30 days, with a mean of 12.7 days post-operatively [26]. Abnormal vital signs and biochemical tests are quite common following the construction of a colorectal anastomosis, and can therefore not be used adequately in the diagnosis of AL. The presentation of AL can vary from abdominal pain and low-grade fever to peritonitis and severe sepsis. This nonspecific course of AL often delays radiologic imaging, and even if this test is undertaken, the diagnoses frequently remain uncertain. Previous research has presented false-negative rates varying from 17-52% for both contrast enemas and computerized tomography (CT) scans [27], resulting in a significant delay of re-intervention [28].

Since a delayed diagnosis of AL is associated with poor outcome [29] and premature re-intervention could lead to a high number of negative re-explorations, one should outweigh the risks of a delayed intervention and the morbidity of re-intervention. Physicians are armed with a restricted number of parameters contributing to risk analysis for the development of AL, with only limited specificity and sensitivity.

### Study Objectives

The objectives of the Predictive Factors of Anastomotic Leakage after Colorectal Surgery (REVEAL) study are twofold. The primary aim of this study is to establish a risk assessment tool for the pre-operative prediction of the development of AL based on a combination of inflammatory, immune-related, and genetic parameters. This algorithm should enable surgeons to make an adequate estimation for every individual patient’s risk of AL, and will eventually aid in the decision-making process regarding the construction of anastomoses and deviating ostomies. In addition, this study aims to provide additional post-operative biomarkers for AL diagnosis at earlier stages, thereby reducing the clinical impact of this feared complication.

## Methods

This is a study protocol for a multicenter, prospective observational study. This study is approved by the Medical Ethical Committee of the Maastricht University Medical Centre. A written informed consent is required from all participating patients and the trial will be conducted in compliance with the rules of *Good Clinical Practice*.

### Study Population

All patients aged >18 years undergoing elective colorectal surgery for colorectal carcinoma, with the construction of an anastomosis, in one of the three participating centers are eligible for inclusion. Patients with large adenomas (large tubular, tubulovillous, or sessile) that cannot be resected radically by means of endoscopy will be included as well, provided that the adenoma is removed surgically with the construction of a colorectal anastomosis. Sample size analysis revealed that 588 consecutive patients have to be included at the outpatient department, prior to surgery. Patients undergoing colorectal surgery for benign conditions, those with permanent stomata without anastomosis, or patients that are unable to give informed consent will be excluded from participation in this study, as well as pregnant patients. If no anastomosis is constructed during surgery, the patient will be withdrawn from the study. Eligibility for inclusion will be determined during a visit at the outpatient department. Upon approval, the patient will receive oral and written information regarding the study, after which he/she will have ample time to reconsider participation. Informed consent will be signed in the presence of the surgeon or researcher.

### Participating Centers

This study will be conducted at Maastricht University Medical Centre (MUMC+, Maastricht, The Netherlands), Zuyderland Medical Centre (Sittard-Geleen and Heerlen, The Netherlands), and Máxima Medical Centre (MMC, Veldhoven, The Netherlands).

### Study Outline

Patients will be admitted to the ward one day prior to surgery, and perioperative care will be performed according to *Enhanced Recovery After Surgery* guidelines for elective colonic surgery [30]. Due to the compelling evidence regarding the detrimental effects of NSAIDs on intestinal wound healing, these drugs will not be administered in this patient population during the perioperative phase. Instead, adequate pain treatment will be provided by means of acetaminophen and opioids if necessary. A *Data Safety Monitoring Board* has been commissioned to evaluate the quality of data collection and monitor patient safety. A web-based system was constructed in order to facilitate the collection of standardized and coded patient data.

The primary end point of this study is post-operative AL, occurring during the first 30 days after surgery, either during hospital admission or following discharge. AL is defined as a communication between the intra- and extra-luminal compartments, resulting from a defect in the integrity of the intestinal wall. Leakage from the suture or staple line from a neorectal reservoir, as well as an abscess near the anastomosis, are also considered leaks [31]. The impact of AL on clinical management is recorded, together with the presence of subclinical (radiological) leaks.

Upon hospital admission, a buccal swab will be performed in order to collect DNA to screen for MBL and COX-2 polymorphisms. In addition, composition of volatile organic compounds (VOCs) in exhaled air will be measured both pre- and post-operatively. Various metabolic processes produce markers that are released into the circulation and, upon passage through the pulmonary system, can be identified as volatile products in exhaled air. The occurrence of (chronic) inflammation and/or oxidative stress can result in the excretion of volatile compounds that generate unique VOC patterns [32]. Furthermore, several baseline characteristics will be acquired prior to surgery, and stool will be collected on three occasions (pre-operatively, post-operatively and at the outpatient department during a control visit) in which the intestinal microbiome can be identified. It has been suggested that the microbiota play an important role in the pathogenesis of AL [33] and that the composition of the intestinal flora can be altered by surgical stress [34]. Plasma will be collected prior to surgery and on post-operative days 1, 3, and 5, in order to determine the concentration of markers for enterocyte damage (eg, I-FABP, citrullin, and calprotectin), transmural ischemia (eg, smooth muscle protein 22), and general inflammation markers (eg, CRP and leukocyte count).

In addition, SNAQ and Malnutrition Universal Screening Tool scores are obtained to assess the nutritional status of the patient, and CT-images are analyzed for total skeletal muscle cross-sectional area in order to distinguish sarcopenic patients from those in good nutritional health [25]. Generalized atherosclerosis will be assessed using abdominal CT-scans, as this is a proposed risk factor for AL [35,36]. Finally, a tissue sample will be obtained from the resected specimen, on which conventional and immunohistochemical staining will be performed.

**Statistical Analyses**

The sample size is calculated with a power analysis and is aimed at our main study outcome, AL. From literature, we know that approximately 10% of all colorectal patients receiving an anastomosis will develop AL [14,37]. The aim of the study is to detect significant and clinically relevant differences between the AL group and the non-AL group. Therefore, we used data from a previous study undertaken by our group to determine sample size [20]. We chose our least significant finding, to avoid the study being underpowered. Based on an effect size of 0.41 (calculated with mean calprotectin levels and SD on day 1), with a power of 0.80 and a 95% confidence interval, the total sample size will be 560, of whom an estimated 51 patients will develop AL. Assuming a 5% dropout rate, the total number of patients that needs to be included in this study is 588.

After data collection, normality will be tested by the D’Agostino-Pearson test. Student’s t-tests will be used for between-group comparisons for continuous data. Dichotomous variables will be compared using Pearson’s chi-squared test. All data will be presented as mean and standard error of the mean. The area under the curve of receiver operating characteristic (ROC) curves will be used to calculate the diagnostic ability of the studied markers predicting AL. The area under the ROC curve is a summary measure of accuracy, lying in the range from 0.5 to 1, with 1 indicating perfect discrimination and 0.5 indicating no discrimination capacity. To determine the most ideal combination of markers, logistic regression analyses will be performed.

## Results

This study has been open for inclusion since August 2015.

## Discussion

Despite important improvements in peri-operative care for CRC surgeries and increased awareness of AL, incidence rates of this dreaded complication have remained stable for several decades. Most strikingly, leakage continues to occur in patients treated under the most expert care, without the presence of any known risk factors [38]. The lack of knowledge regarding the pathophysiological process of AL and the process of normal intestinal healing hampers the development of novel predictive and preventive methods. AL has significant impacts on morbidity and mortality, quality of life, and health care costs, and is suggested to negatively interfere with oncological prognoses [5,6,7,8]. Although large numbers of clinical trials have identified important patient-related and technical factors that aid in the prediction of AL [38,39], the search for a predictive biomarker for AL has hitherto remained unsuccessful. Almost all previously conducted studies reported a single risk factor for the development of AL in CRC patients undergoing surgery. The multi-modal design of the current study enables us to bypass the limitations encountered by previous studies.

In order to minimize the potentially life threatening clinical consequences of a leak, some surgeons opt to perform a deviating ostomy directly following the initial operation [41]. Although it is generally accepted that the presence of a colostoma or ileostoma reduces the sequelae of AL and the need for reoperation in case of a leak [42,43], a decrease in incidence of post-operative mortality has not yet been proven [42-46]. The presence of selection bias, in which an ostomy is performed in high-risk patients, should be considered when interpreting these results. Surgeons perform elective deviating ostomy in approximately 70% of cases of low rectal carcinoma, of which a significant percentage (19-40%) will never have their temporary ostomy reversed [47,48]. Possible benefits of a deviating stoma should be weighed against stoma-related morbidity, the impact on quality of life, and the mortality rates after stoma closure [49,50]. An adequate pre-operative risk analysis could aid surgeons and their patients in the decision-making process regarding the construction of temporary ostomies. The successful implementation of risk assessment tools would have a positive influence on morbidity and mortality rates, duration of hospital stays, and number of readmissions, re-interventions, and admissions to the ICU, leading to a significant increase in quality of life for the general patient population [38]. Anastomoses are also constructed in gastrointestinal surgery for other purposes than malignancies, such as inflammatory bowel disease or diverticulitis [51,52]. We have chosen to exclude these patients since their inflammatory status can be a confounding factor in the early detection and/or risk assessment of AL [53-55].

This study outline is based on the hypothesis that AL is partly due to patient-derived factors such as a derailed immune response, genetic predisposition, and a deficient microbiome composition, and that the clinical course can be further influenced by surgical stress, ischemia, and a compromised systemic response. This study aims at broadening our understanding of the pathophysiological process of AL by introducing novel biomarkers of intestinal damage and function, and to decrease the clinical burden of AL by both individual pre-operative risk assessment and early post-operative detection in the future.

## Abbreviations

AL: anastomotic leakage
COX-2: cyclo-oxygenase 2
CRC: colorectal cancer
CRP: C-reactive protein
CT: computerized tomography
I-FABP: intestinal fatty acid binding protein
MBL: mannose-binding lectin
NSAIDs: non-steroidal anti-inflammatory drugs
OR: odds ratio
REVEAL: Predictive Factors of Anastomotic Leakage after Colorectal Surgery
ROC: receiver operating characteristic
SNAQ: short nutritional assessment questionnaire
VOC: volatile organic compound
